# Krempfielins N–P, New Anti-Inflammatory Eunicellins from a Taiwanese Soft Coral *Cladiella krempfi*

**DOI:** 10.3390/md12021148

**Published:** 2014-02-21

**Authors:** Yan-Ning Lee, Chi-Jen Tai, Tsong-Long Hwang, Jyh-Horng Sheu

**Affiliations:** 1 Department of Marine Biotechnology and Resources, National Sun Yat-sen University, Kaohsiung 80424, Taiwan; E-Mails: jennyyanningl@yahoo.com.tw (Y.-N.L.); jean801023@hotmail.com (C.-J.T.); 2 Graduate Institute of Natural Products, Chang Gung University, Taoyuan 33302, Taiwan; E-Mail: htl@mail.cgu.edu.tw; 3 Division of Marine Biotechnology, Asia-Pacific Ocean Research Center, National Sun Yat-sen University, Kaohsiung 80424, Taiwan; 4 Department of Medical Research, China Medical University Hospital, China Medical University, Taichung 40402, Taiwan; 5 Graduate Institute of Natural Products, Kaohsiung Medical University, Kaohsiung 80708, Taiwan

**Keywords:** *Cladiella krempfi*, eunicellin-type diterpenoid, anti-inflammatory agent, elastase

## Abstract

Three new eunicellin-type diterpenoids, krempfielins N–P (**1**–**3**), were isolated from a Taiwanese soft coral *Cladiella krempfi*. The structures of the new metabolites were elucidated by extensive spectroscopic analysis and by comparison with spectroscopic data of related known compounds. Compound **3** exhibited activity to inhibit superoxide anion generation. Both **1 **and**3**, in particular **1**, were shown to display significant anti-inflammatory activity by inhibiting the elastase release in FMLP/CB-induced human neutrophils.

## 1. Introduction

**Chart 1:**
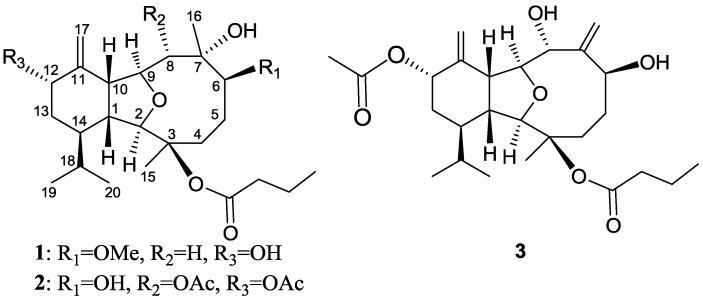
Structures of metabolites **1**–**3**.

Soft corals have been known to be rich sources of terpenoid metabolites [1]. For the purpose of discovering bioactive agents from marine organisms, we have previously investigated the chemical constituents and reported a series of bioactive natural products from Taiwanese soft corals [2,3,4,5]. In recent studies a series of bioactive eunicellin-based diterpenoids, have been isolated from the soft corals of the genera* Cladiella*, *Klysum* and *Litophyton* sp. [6,7,8,9,10,11,12,13,14]. The soft coral *Cladiella krempfi* has been found to produce several types of metabolites including eunicellin-type diterpenoids [15,16,17] and pregnane-type steroids [18,19]. Our previous chemical investigation of the Formosan soft coral *Cladiella krempfi* also resulted in the isolation of a series of new eunicellin-type diterpenoids, krempfielins A–M [20,21,22]. In this paper, we further report the discovery of three new eunicellin-based diterpenoids, krempfielins N–P (**1**–**3**) (Chart 1 and Supplementary Figures S1–S9). The ability of these compounds to inhibit the superoxide anion generation and elastase release in FMLP/CB-induced human neutrophils was also evaluated. The results showed that compound **3** could inhibit superoxide anion generation while **1** and **3**, especially **1**, effectively inhibited the generation of the elastase release in FMLP/CB-induced human neutrophils.

## 2. Results and Discussion

**Figure 1:**
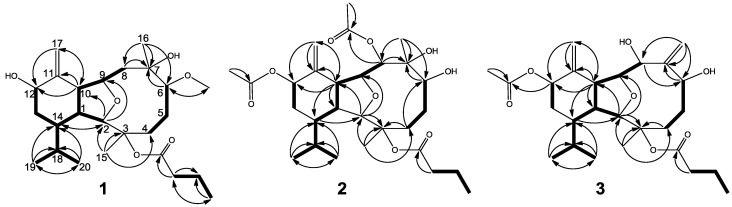
Selected ^1^H−^1^H COSY (▬) and HMBC (→) correlations of **1**, **2** and **3**.

The new metabolite krempfielin N (**1**) showed the molecular ion peak [M + Na]^+^ at *m*/*z* 461.2882 in the HRESIMS and established a molecular formula of C_25_H_42_O_6_, implying five degrees of unsaturation. The IR absorptions bands at ν_max_ 3445 and 1733 cm^−1^ revealed the presence of hydroxy and ester carbonyl functionalities. The ^13^C NMR spectrum measured in CDCl_3_ showed signals of 25 carbons (Table 1) which were assigned by the assistance of the DEPT spectrum to six methyls (including one oxgenate methyl δ_C_ 57.0), six sp^3^ methylenes, one sp^2^ methylene, eight sp^3^ methines (including four oxymethines), four quaternary carbons (including one ester carbonyl). The NMR spectroscopic data of **1** (Table 1 and Table 2) showed the presence of one 1,1-disubstituted double bond (δ_C_ 112.5 CH_2_ and 148.0 C; δ_H_ 5.03 s, and 4.86 s), one methoxy group (δ_H_ 3.34, 3H, s) and one *n*-butyryloxy group (δ_C_ 172.3 C; 37.4 CH_2_; 18.4 CH_2_; and 13.7 CH_3_; δ_H_ 2.30 m, 2H; 1.67 m, 2H; and 0.98 t, 3H, *J* = 7.6 Hz). Therefore, taking account of the two degrees of unsaturation from double bonds, it was suggested that **1** should be a tricyclic compound from the remaining three degrees of unsaturation. The ^1^H–^1^H COSY and HMBC correlations (Figure 1) were further used for establishing the molecular skeleton of **1**. The COSY experiment assigned three isolated consecutive proton spin systems. Above evidences and the analysis of HMBC spectrum (Figure 1) suggested that **1** is an eunicellin-based diterpenoid. Furthermore, the two hydroxy groups attaching at C-7 and C-12 were confirmed by the HMBC correlations from one methyl (δ_H_ 1.12 s, H-16) and one oxymethine (δ_H_ 4.12 m, H-6) to the oxygenated quaternary carbon appearing at δ 75.8 (C-7), and one methine (δ_H_ 2.91 t, H-10) and one proton of H_2_-17 (δ_H_ 5.03 s) to the oxymethine carbon appearing at δ 71.0 (C-12). Thus, the remaining one *n*-butyryloxy group had to be positioned at C-3, an oxygen-bearing quaternary carbon resonating at δ 86.5 ppm. On the basis of above analysis, the planar structure of **1** was established. The stereochemistry of **1** was finally confirmed by the very similar NOE correlations of both **1** and krempfielin L [22].

**Table 1:** ^13^C NMR data for compounds **1**–**3**.

	1 ^a^	2 ^b^	3 ^a^
	δ_C_	δ_C_	δ_C_
1	44.4, CH ^c^	45.1, CH	43.2, CH ^c^
2	91.0, CH	91.5, CH	90.7, CH
3	86.5, C	85.7, C	84.5, C
4	36.0, CH_2_	35.7, CH_2_	28.7, CH_2_
5	26.7, CH_2_	28.9, CH_2_	35.4, CH_2_
6	89.7, CH	77.5, CH	67.0, CH
7	75.8, C	79.4, C	152.5, C
8	45.2, CH_2_	79.0, CH	77.2, CH
9	80.0, CH	79.4, CH	85.0, CH
10	51.4, CH	50.1, CH	46.6, CH
11	148.0, C	143.6, C	141.4, C
12	71.0, CH	73.5, CH	73.2, CH
13	30.7, CH_2_	29.2, CH_2_	29.0, CH_2_
14	36.6, CH	37.2, CH	36.9, CH
15	23.2, CH_3_	23.1, CH_3_	22.1, CH_3_
16	23.6, CH_3_	18.0, CH_3_	118.1, CH_2_
17	112.5, CH_2_	115.1, CH_2_	119.4, CH_2_
18	28.8, CH	28.6, CH	26.9, CH
19	16.0, CH_3_	15.6, CH_3_	15.3, CH_3_
20	21.8, CH_3_	21.7, CH_3_	21.6, CH_3_
3- *n*-butyrate	172.3, C	173.0, C	172.5, C
	37.4, CH_2_	36.7, CH_2_	37.4, CH_2_
	18.4, CH_2_	18.5, CH_2_	18.5, CH_2_
	13.7, CH_3_	13.5, CH_3_	13.6, CH_3_
6-OMe	57.0, CH_3_		
8-OAc		170.7, C	
		21.4, CH_3_	
12-OAc		170.2, C	170.1, C
		21.6, CH_3_	21.7, CH_3_

^a^^ 13^C spectra recorded at 100 MHz in CDCl_3_;^ b^^ 13^C spectra recorded at 125 MHz in CDCl_3_; ^c ^Deduced from DEPT.

**Table 2:** ^1^H NMR data for compounds **1**–**3**.

	1 ^a^	2 ^b^	3 ^a^
	δ_H_	δ_H_	δ_H_
1	2.25 m	2.28 dd (10.0, 7.0)	2.26 m
2	3.70 br s	3.67 br s	3.84 br s
3			
4	1.86 m, 2.64 m	1.85 m, 2.66 m	1.68 m, 2.66 m
5	1.33 m, 1.65 m	1.49 m, 1.65 m	1.76 m
			2.21 m
6	4.12 m	4.66 d (6.5)	4.75 dd (10.8, 4.4) ^d^
7			
8	1.82 m	5.19 d (10.0)	4.20 s
9	4.53 m	4.31 dd (10.0, 6.5)	4.43 d (10.8)
10	2.91 t (6.4) ^c^	3.38 dd (7.0, 7.0)	2.90 dd (10.8, 8.4)
11			
12	4.39 s	5.43 dd (4.0, 3.0)	5.49 t (2.8)
13	1.36 m, 1.86 m	1.37 m, 1.93 m	1.30 m, 1.98 m
14	1.86 m	1.70 m	1.71 m
15	1.45 s	1.45 s	1.65 s
16	1.12 s	1.08 s	5.23 s, 5.55 s
17	4.86 s, 5.03 s	4.84 s, 5.10 s	4.96 d (1.6)
			5.27 d (1.6)
18	1.81 m	1.80 m	1.96 m
19	0.82 d (7.6)	0.80 d (7.0)	0.75 d (6.8)
20	0.99 d (7.2)	0.95 d (7.0)	0.95 d (6.8)
3- *n*-butyrate	2.30 m	2.60 m, 2.50 m	2.12 m
	1.67 m	1.67 m	1.58 m
	0.98 t (7.6)	1.00 t (7.5)	0.92 t (7.6)
6-OMe	3.34 s		
8-OAc		2.07 s	
12-OAc		2.08 s	2.05 s

^a^^ 1^H spectra recorded at 400 MHz in CDCl_3_;^ b^^ 1^Hspectra recorded at 500 MHz in CDCl_3_;^ c^* J* values (Hz) in parentheses.

**Figure 2:**
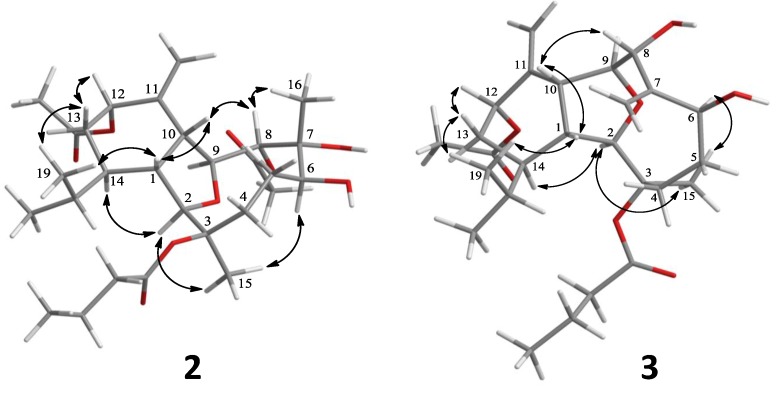
Key NOESY correlations for **2** and **3**.

Krempfielin O (**2**) was shown by HRESIMS to possess the molecular formula C_2__8_H_4__4_O_9_ (*m*/*z* 547.2880 [M + Na]^+^). The NMR spectroscopic data of **2** (Table 1 and Table 2) showed the presence of two acetoxy groups (δ_H_ 2.07, s and 2.08, s, each 3H; and δ_C_ 170.7, C and 170.2, C; 21.4, CH_3_ and 21.6, CH_3_), and an *n*-butyryloxy group (δ_H_ 2.60 m and 2.50 m, each 1H; 1.67 m, 2H and 1.00 t, 3H, *J* = 7.5 Hz; and δ_C_ 173.0, C; 36.7, CH_2_; 18.5, CH_2_ and 13.5, CH_3_). As demonstrated by the HMBC correlation from oxymethine proton H-8 (δ 5.19) to the ester carbonyl carbon appearing at δ_C_ 170.7 (Figure 1), one acetoxy group was positioned at C-8. The position of an *n*-butyryloxy group at C-3 was established by NOE interaction between the methylene protons (δ 1.67) of *n*-butyryloxy group with H-5 (δ 1.49). The remaining one acetoxy group was thus positioned at C-12. The relative configuration of **2** was further confirmed by NOE correlations (Figure 2).

The related metabolite, krempfielin P (**3**), had a molecular formula of C_26_H_40_O_7_ as indicated by the HRESIMS (*m*/*z* 487.2675, [M + Na]^+^) and NMR data (Table 1 and Table 2). The ^13^C NMR spectrum of **3** revealed the appearance of two ester carbonyls (δ_C_ 172.5 and 170.1), which were correlated with one methylene (δ_H_ 2.12 m, 2H; and δ_C_ 37.4) of an *n*-butyrate and the methyl (δ_H_ 2.05 s, 3H; δ_C_ 21.7CH_3_) of an acetate group, respectively. The planar structure of **3** was determined by ^1^H–^1^H COSY and HMBC correlations (Figure 1). Comparison of the NMR data of **3** with those of the compound krempfielin A [20] revealed that the only difference is the replacement of one methyl and one hydroxy group at C-7 in krempfielin A by the substitution of one olefinic methylene (δ_C_ 118.1, CH_2_; δ_H_ 5.55, s and 5.23, s) in **3**. The placement of one *n*-butyryloxy group and one acetoxy group at C-3 and C-12, respectively was established by comparison of the spectroscopic data with those of krempfielin A. The relative configuration of **3** was mostly determined to be the same as that of krempfielin A by comparison of the chemical shifts of both compounds and was further confirmed by NOE correlations (Figure 2). 

Recently, we discovered several eunicellins showed anti-inflammatory activity by significantly inhibiting superoxide anion generation and elastase release in human neutrophiles induced by *N*-formyl-methionyl-leucyl-phenylalanine/cytochalasin B (FMLP/CB) [22,23]. The same*in vitro* anti-inflammatory effects of the diterpenoids **1**–**3** also were tested in this study (Table 3). At a concentration of 10 µM, **1** and **2** could not significantly reduce the generation of superoxide anion, however, **3** inhibited 23.32% ± 5.88% generation of superoxide anion, relative to the control cells stimulated with FMLP/CB only. At the same concentration, all of **1**–**3** were found to show anti-inflammatory activity by inhibiting the elastase release. Compound **1** displayed significant inhibition (73.86% ± 14.18%) at this concentration with IC_50_ of 4.94 ± 1.68 µM in this assay.

**Table 3:** Effect of pure compounds on elastase release in *N*-formyl-methionyl-leucyl-phenylalanine/cytochalasin B (FMLP/CB)-induced human neutrophils.

Compound	Elastase		
	Inhibition (%)		IC_50_ (µM)
**1**	73.86 ± 14.18	**	4.94 ± 1.68
**2**	13.33 ± 3.56	*	>10
**3**	35.54 ± 3.17	***	>10

Percentage of inhibition (%) was measured at 10 µM; results are presented as mean ± S.E.M. (*n* = 3 or 4); * p < 0.05, ** *p* < 0.01 and *** p < 0.001 compared with the control value.

## 3. Experimental Section

### 3.1. General Experimental Procedures

Melting point was determined using a Fisher-Johns melting point apparatus. Optical rotations were measured on a JASCO P-1020 polarimeter. IR spectra were recorded on a JASCO FT/IR-4100 infrared spectrophotometer. ESIMS were obtained with a Bruker APEX II mass spectrometer. The NMR spectra were recorded either on a Varian UNITY INOVA-500 FT-NMR and a Varian 400MR FT-NMR. Silica gel (Merck, Darmstadt, Germany, 230–400 mesh) was used for column chromatography. Precoated silica gel plates (Merck, Darmstadt, Germany, Kieselgel 60 F-254, 0.2 mm) were used for analytical thin layer chromatography (TLC). High performance liquid chromatography was performed on a Hitachi L-7100 HPLC apparatus with an octadecylsilane (ODS) column (250 × 21.2 mm, 5 µm).

### 3.2. Animal Material

*C. krempfi* was collected by hand using scuba off the coast of Penghu islands of Taiwan in June 2008, at a depth of 5–10 m, and stored in a freezer until extraction. A voucher sample (specimen No. 200806CK) was deposited at the Department of Marine Biotechnology and Resources, National Sun Yat-sen University*.*

### 3.3. Extraction and Separation

The octocoral (1.1 kg fresh wt) was collected and freeze-dried. The freeze-dried material was minced and extracted exhaustively with EtOH (3 × 10 L). The EtOH extract of the frozen organism was partitioned between CH_2_Cl_2_ and H_2_O. The CH_2_Cl_2_-soluble portion (14.4 g) was subjected to column chromatography on silica gel and eluted with EtOAc in *n*-hexane (0%–100% of EtOAc, stepwise) and then further with MeOH in EtOAc with increasing polarity to yield 41 fractions. Fraction 31, eluted with *n*-hexane–EtOAc (1:10), was rechromatoraphed over a silica gel open column using *n*-hexane–acetone (3:1) as the mobile phase to afford eight subfractions (A1–A8). Subfraction A4 was repeatedly separated by reverse phase HPLC (CH_3_CN–H_2_O, 0.8:1 to 1:1) to afford compound **1** (3.2 mg). Subfraction A5 separated by reverse phase HPLC (CH_3_CN–H_2_O, 1:1 to 1:1.6) to afford compound **2** (1.2 mg). Subfraction A6 by reverse phase HPLC (CH_3_CN–H_2_O, 1:1.5) to afford compound **3** (3.9 mg). 

#### 3.3.1. Krempfielin N (1)

Colorless oil; 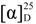 = +27.3 (*c* 0.91, CHCl_3_); IR (neat) ν_max_ 3445, 2961, 1733, 1457, 1370, 1180, and 1084 cm^−1^; ^13^C and ^1^H NMR data, see Table 1 and Table 2; ESIMS *m*/*z* 461 [M + Na]^+^; HRESIMS *m*/*z* 461.2882 [M + Na]^+^ (calcd. for C_25_H_42_O_6_Na, 461.2879).

#### 3.3.2. Krempfielin O (2)

Colorless oil; 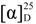 = −56.7 (*c* 0.3, CHCl_3_); IR (neat) ν_max_ 3461, 2960, 1735, 1464, 1372, 1238, 1177, 1076, and 1026 cm^−1^; ^13^C and ^1^H NMR data, see Table 1 and Table 2; ESIMS *m*/*z* 547 [M + Na]^+^; HRESIMS *m*/*z* 547.2880 [M + Na]^+^ (calcd. for C_28_H_44_O_9_Na, 547.2883).

#### 3.3.3. Krempfielin P (3)

Colorless oil; 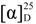 = +13.1 (*c* 3.8, CHCl_3_); IR (neat) ν_max_ 3419, 2959, 1733, 1437, 1371, 1237, 1182, and 1072 cm^−1^; ^13^C and ^1^H NMR data, see Table 1 and Table 2; ESIMS *m*/*z* 487 [M + Na]^+^; HRESIMS *m*/*z* 487.2675 [M + Na]^+^ (calcd for C_26_H_40_O_7_Na, 487.2672).

### 3.4. *In Vitro* Anti-Inflammatory Assay—Superoxide Anion Generation and Elastase Release by Human Neutrophils

Human neutrophils were obtained by means of dextran sedimentation and Ficoll centrifugation. Measurements of superoxide anion generation and elastase release were carried out according to previously described procedures [24,25]. LY294002, a phosphatidylinositol-3-kinase inhibitor, was used as a positive control for inhibition of superoxide anion generation and elastase release with IC_50_ values of 1.88 ± 0.45 and 4.12 ± 0.92 µM, respectively. Briefly, superoxide anion production was assayed by monitoring the superoxide dismutase-inhibitable reduction of ferricytochrome c. Elastase release experiments were performed using MeO-Suc-Ala-Ala-Pro-Val-*p*-nitroanilide as the elastase substrate.

## 4. Conclusions

New eunicellin-based diterpenoids were isolated from the soft coral *Cladiella krempfi.* Compounds **1** and **3**, especially **1**, could significantly inhibit the release of elastase in FMLP/CB-induced human neutrophils, and **3** inhibited 23% generation of superoxide anion. Thus, compounds **1** and **3** are promising anti-inflammatory agents and may warrant further biomedical investigation.

## Supplementary Files

Supplementary File 1 Supplementary Information (PDF, 464 KB)
