# Supplementary material for: Krempfielins N–P, New Anti-Inflammatory Eunicellins from a Taiwanese Soft Coral Cladiella krempfi
Source: Mar Drugs. 2014 Feb 21;12(2):1148–56. doi: 10.3390/md12021148 (PMC3944535; doi:10.3390/md12021148)
Supplement: Supplementary File 1 — Supplementary Information (PDF, 464 KB) [file marinedrugs-12-01148-s001.pdf]

Supplementary Information

Figure S1. ^{1}H NMR spectrum of **1** in $CDCl_3$ at 400 MHz.

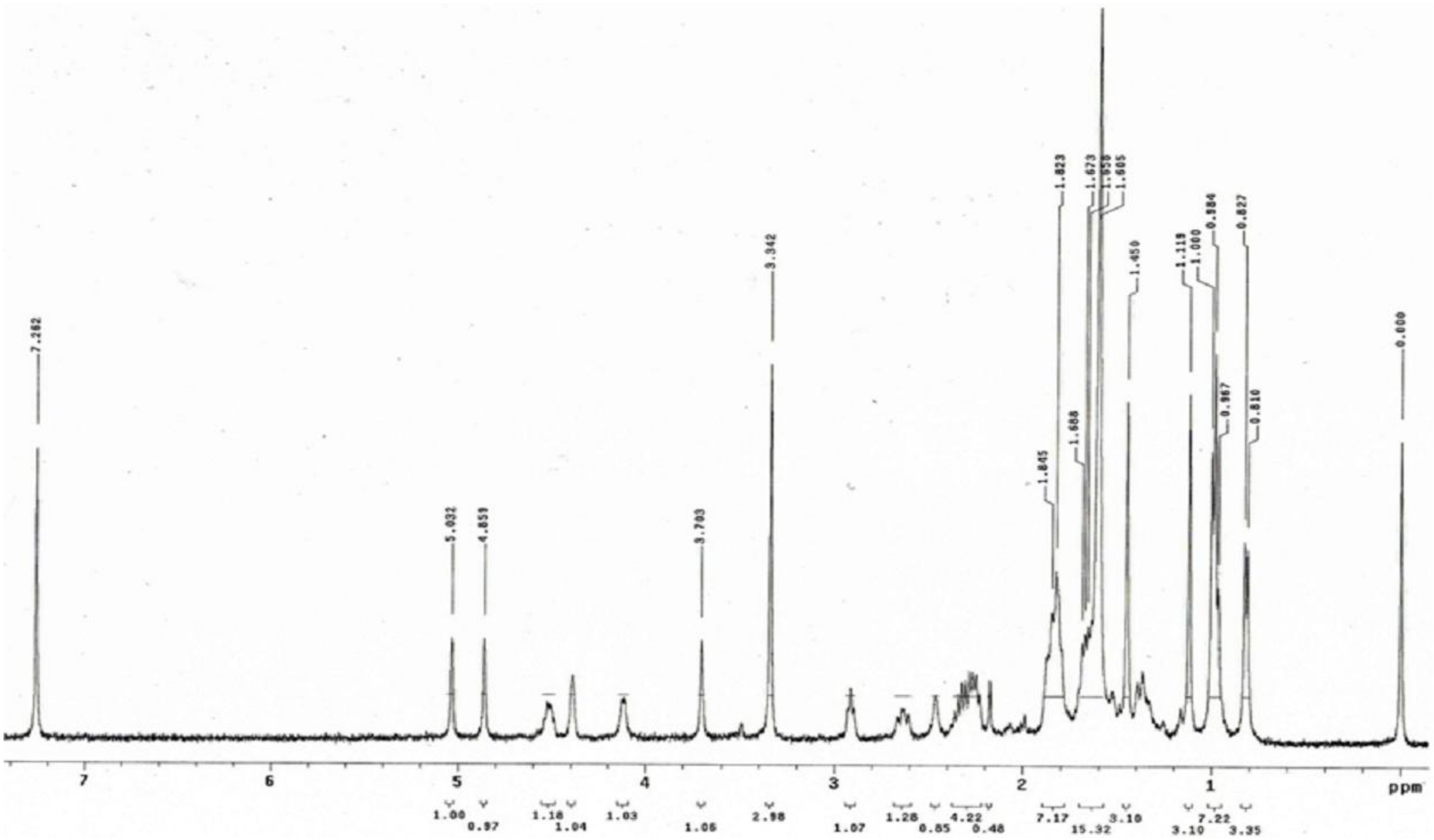

Figure S2. ^{13}C NMR spectrum of **1** in $CDCl_3$ at 100 MHz.

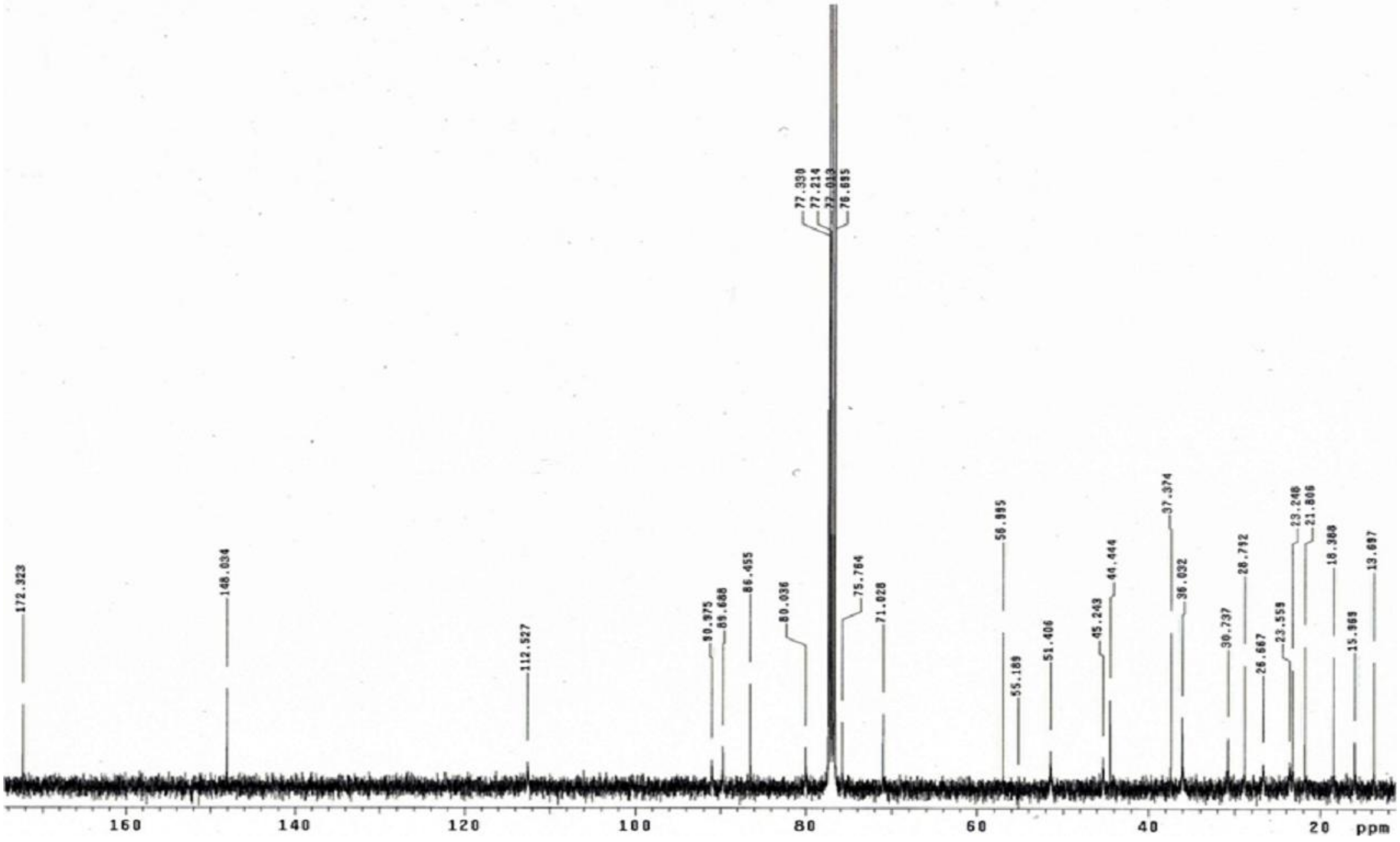

Figure S3. HRESIMS spectrum of **1**.

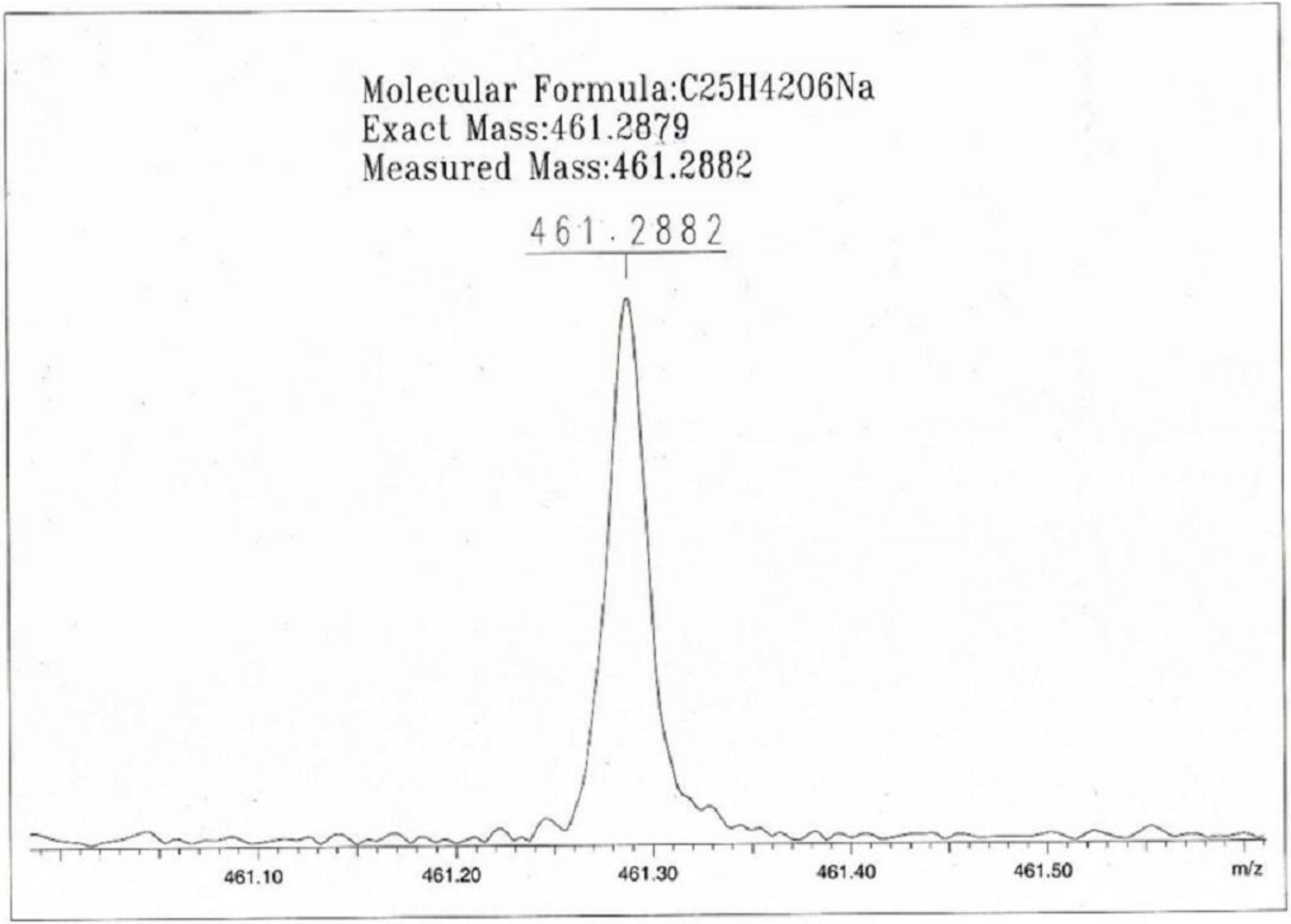

Figure S4. ^{1}H NMR spectrum of **2** in $CDCl_3$ at 500 MHz.

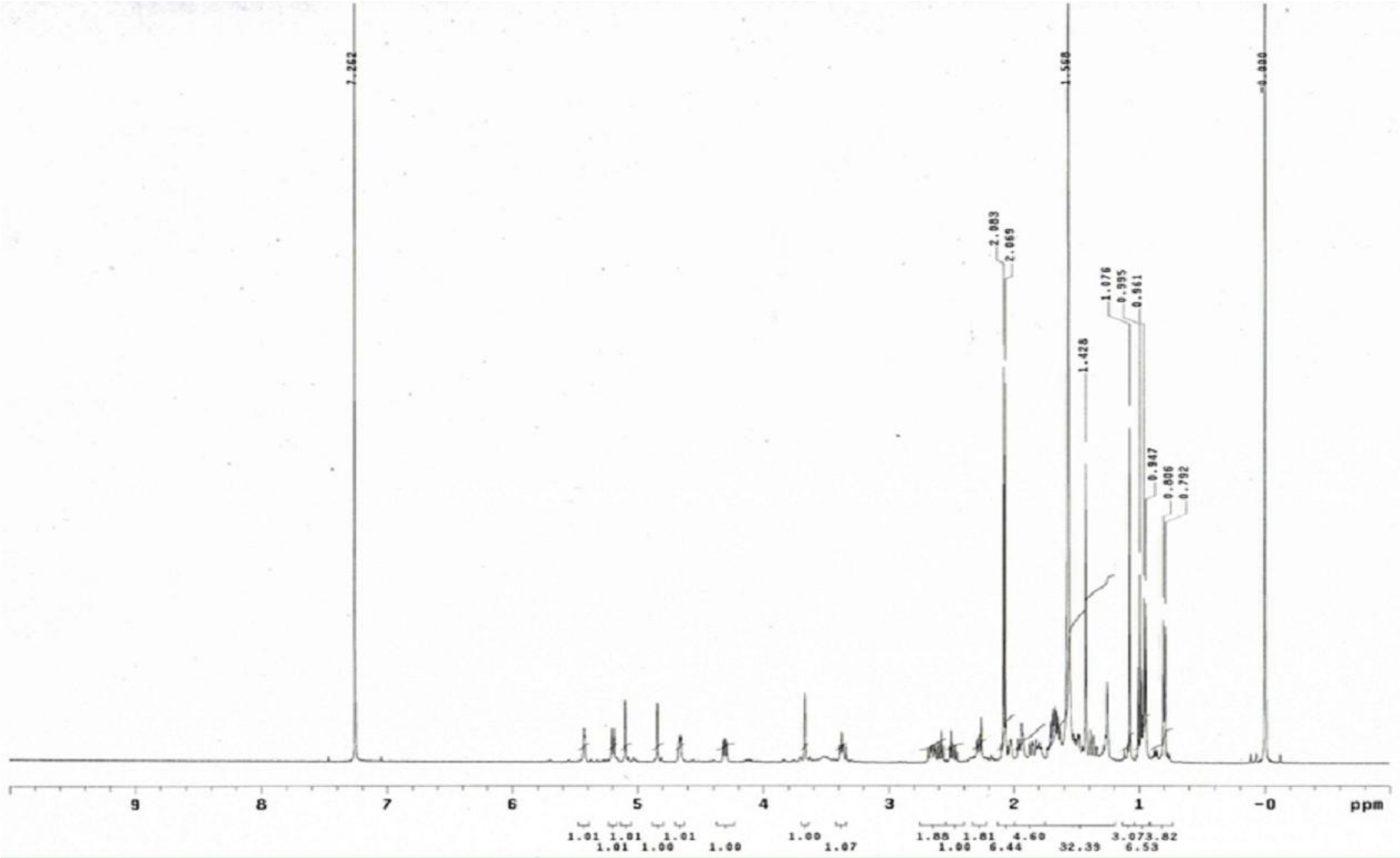

Figure S5. ^{13}C NMR spectrum of **2** in $CDCl_3$ at 125 MHz.

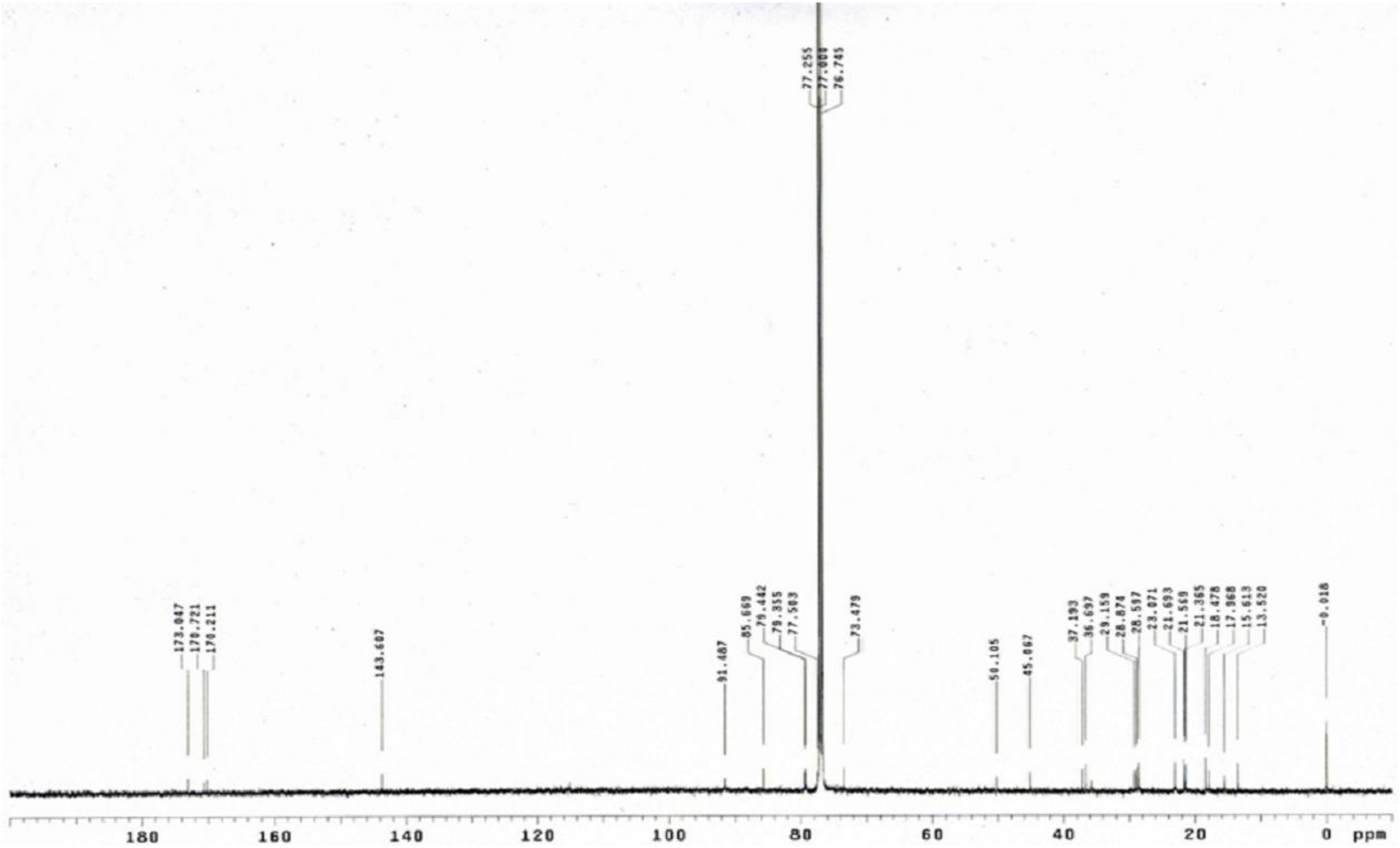

Figure S6. HRESIMS spectrum of **2**.

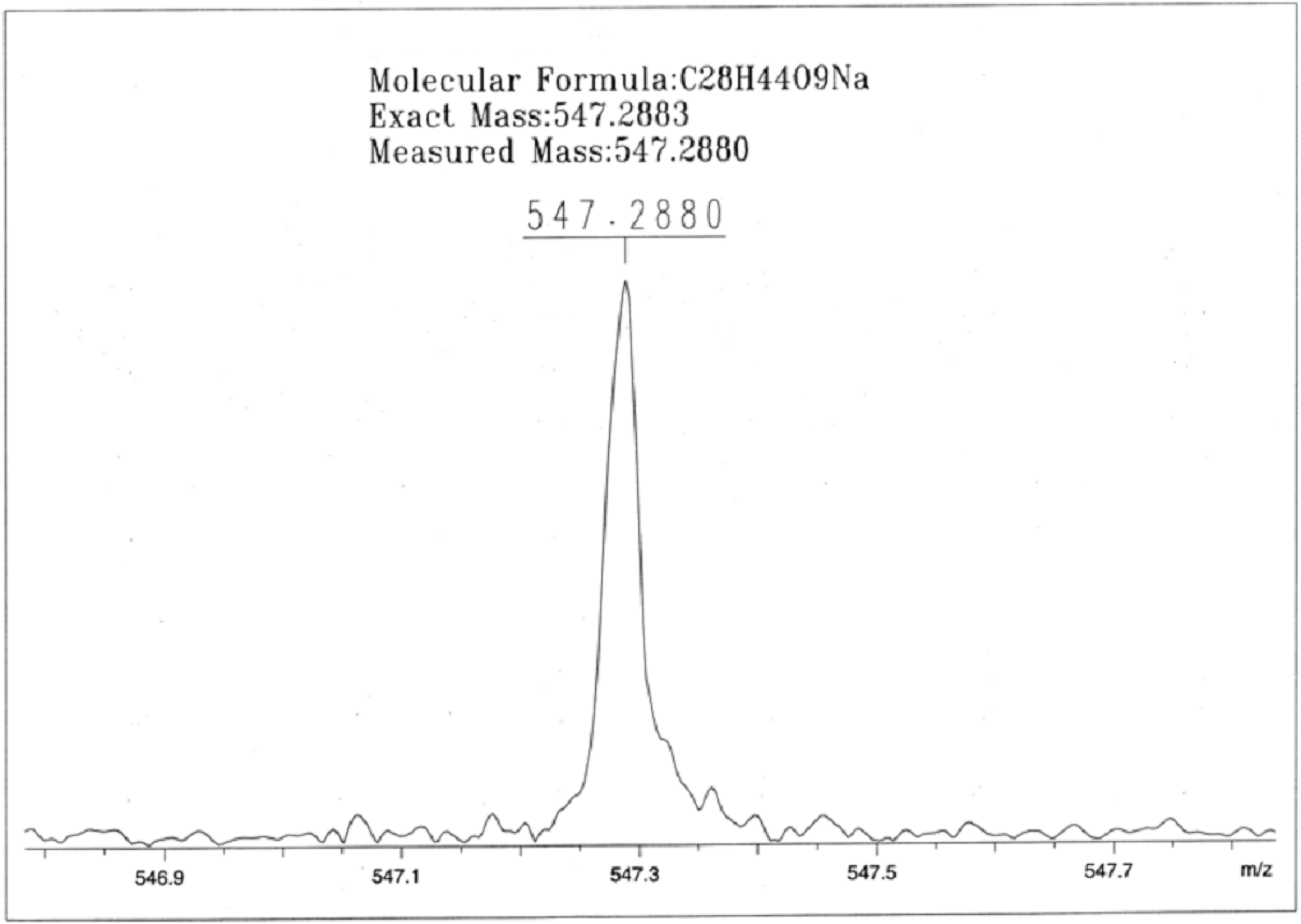

Figure S7. ^{1}H NMR spectrum of **3** in $CDCl_3$ at 400 MHz.

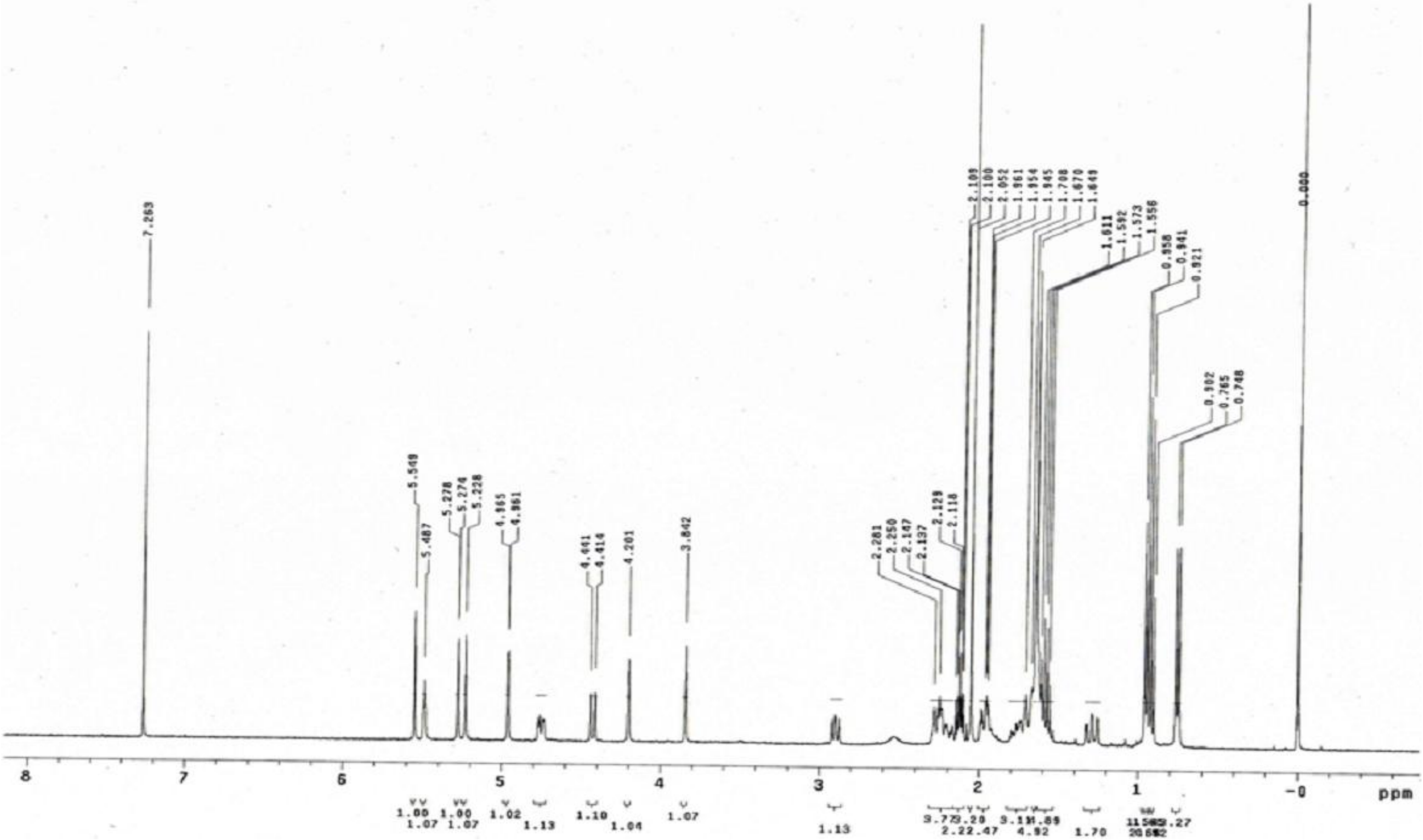

Figure S8. ^{13}C NMR spectrum of **3** in $CDCl_3$ at 100 MHz.

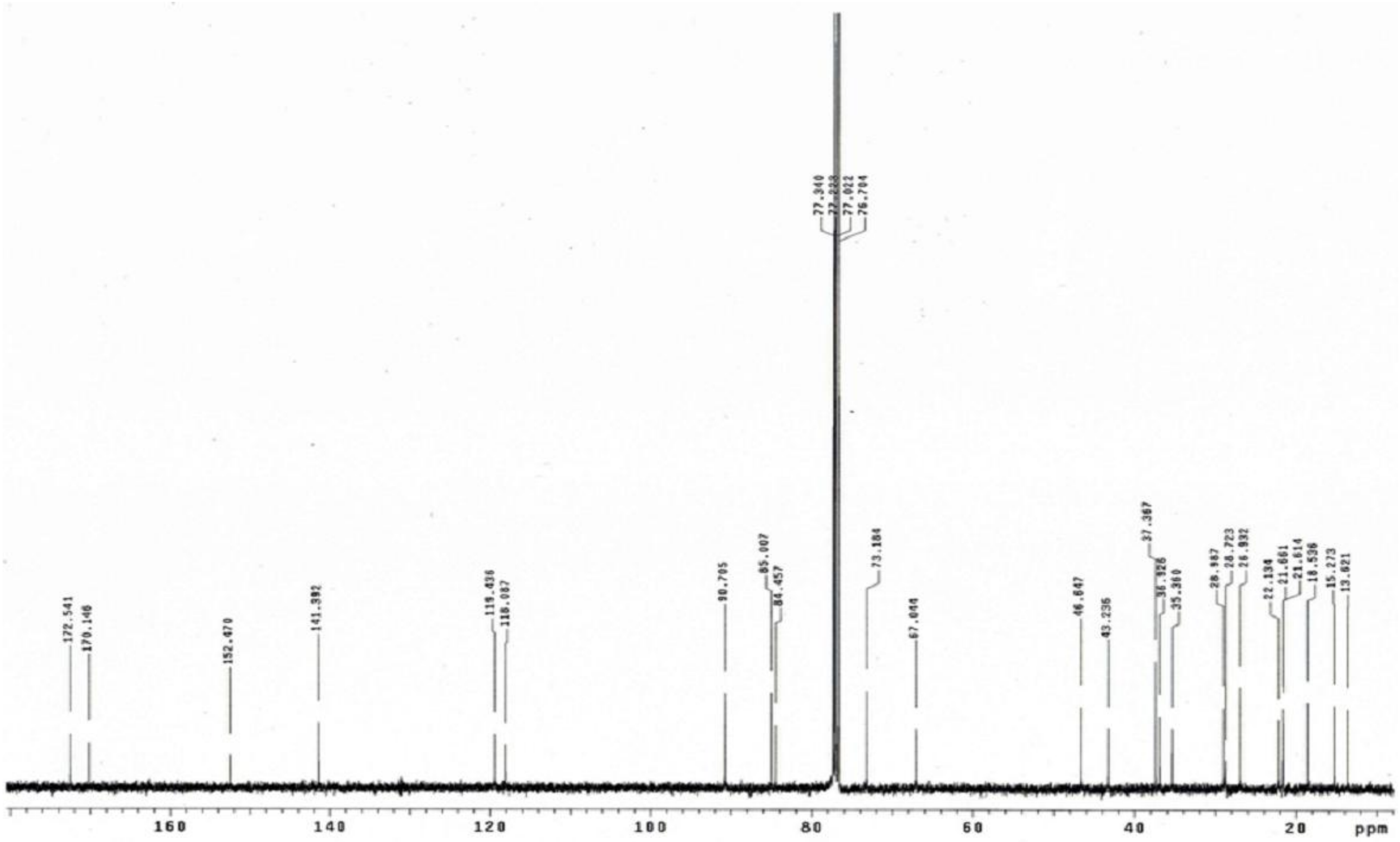

Figure S9. HRESIMS spectrum of **3**.

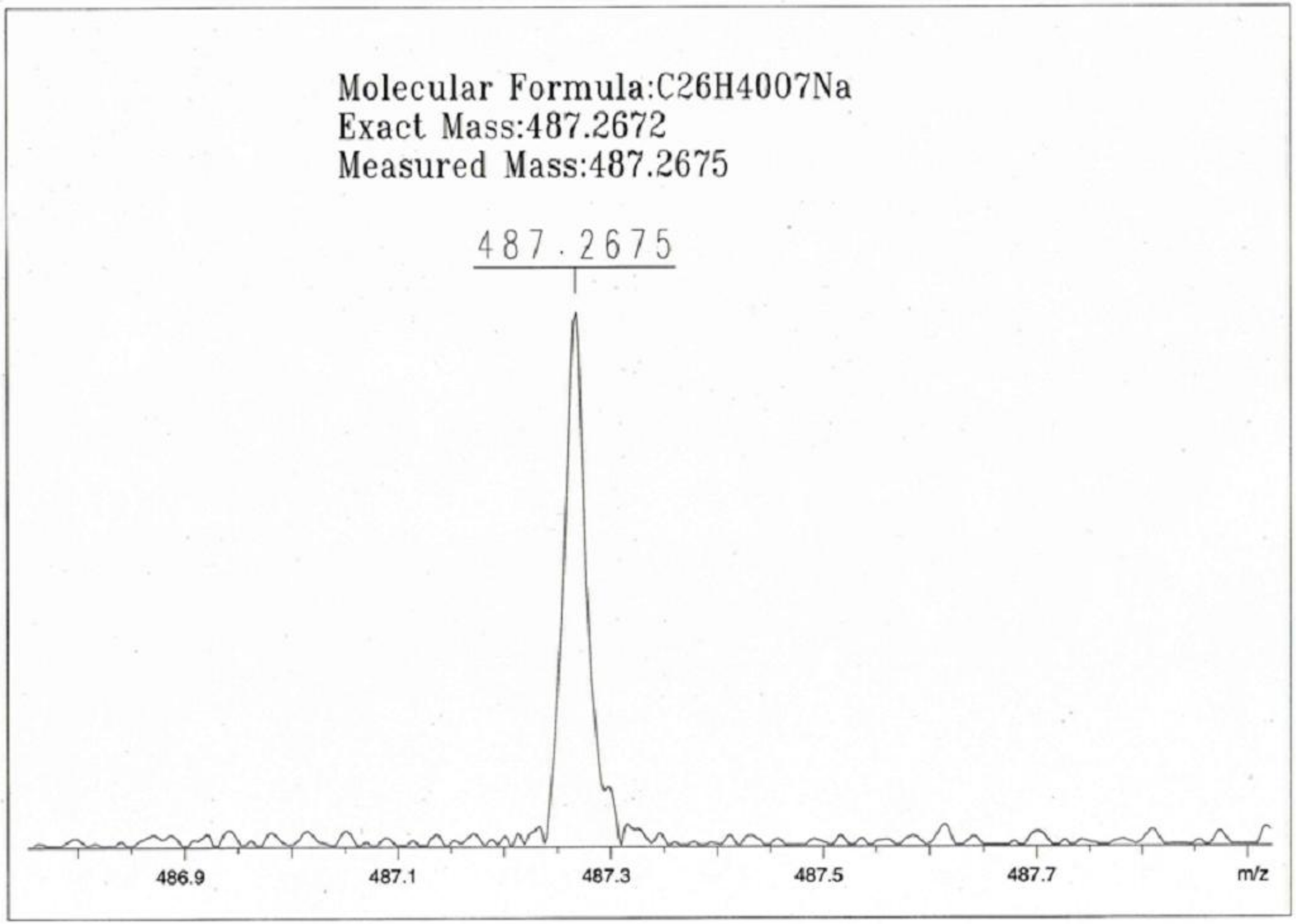

© 2014 by the authors; licensee MDPI, Basel, Switzerland. This article is an open access article distributed under the terms and conditions of the Creative Commons Attribution license (http://creativecommons.org/licenses/by/3.0/).
